# TCTP is Essential for Cell Proliferation and Survival during CNS Development

**DOI:** 10.3390/cells9010133

**Published:** 2020-01-06

**Authors:** Sung-Ho Chen, Chin-Hung Lu, Ming-Jen Tsai

**Affiliations:** 1 Department of Pharmacology, College of Medicine, Tzu Chi University, Hualien 97004, Taiwan; 2 Master Program in Pharmacology and Toxicology, College of Medicine, Tzu Chi University, Hualien 97004, Taiwan; Lu721112@gmail.com; 3 Department of Emergency Medicine, Ditmanson Medical Foundation Chia-Yi Christian Hospital, Chiayi City 600, Taiwan; tshi33@gmail.com

**Keywords:** apoptosis, conditional knockout mice, development, Nestin-cre, neurogenesis, neuronal progenitor cells, perinatal death, proliferation, TCTP

## Abstract

Translationally controlled tumor-associated protein (TCTP) has been implicated in cell growth, proliferation, and apoptosis through interacting proteins. Although TCTP is expressed abundantly in the mouse brain, little is known regarding its role in the neurogenesis of the nervous system. We used *Nestin-cre*-driven gene-mutated mice to investigate the function of TCTP in the nervous system. The mice carrying disrupted TCTP in neuronal and glial progenitor cells died at the perinatal stage. The *Nestin^C^^re^*^/+^; *TCTP^f^*^/*f*^ pups displayed reduced body size at postnatal day 0.5 (P0.5) and a lack of milk in the stomach compared with littermate controls. In addition to decreased cell proliferation, terminal deoxynucleotidyl transferase-mediated dUTP nick end-labeling (TUNEL) and caspase assay revealed that apoptosis was increased in newly committed TCTP-disrupted cells as they migrated away from the ventricular zone. The mechanism may be that the phenotype from specific deletion of TCTP in neural progenitor cells is correlated with the decreased expression of cyclins D2, E2, Mcl-1, Bcl-xL, hax-1, and Octamer-binding transcription factor 4 (Oct4) in conditional knockout mice. Our results demonstrate that TCTP is a critical protein for cell survival during early neuronal and glial differentiation. Thus, enhanced neuronal loss and functional defect in Tuj1 and doublecortin-positive neurons mediated through increased apoptosis and decreased proliferation during central nervous system (CNS) development may contribute to the perinatal death of *TCTP* mutant mice.

## 1. Introduction

Translationally controlled tumor-associated protein (TCTP) is a highly conserved and abundantly expressed protein that has been implicated in both cell growth and the human acute allergic response. TCTP is widely expressed in many tissues and cell types [1], and its protein levels are highly regulated in response to a wide range of extracellular signals and cellular conditions [2]. TCTP has been shown to interact with tubulin [3], Na^+^, K^+^-ATPase [4], mammalian Plk [5], translation elongation factors eEF1A and eEF1Bbeta [6,7], TSAP6 [8], Mcl-1 [9], Bcl-XL [10], vitamin D3 receptor (VDR) [11], and p53 [12] for cell cycle regulation, protein synthesis, and antiapoptosis.

TCTP is also known as a histamine-releasing factor that stimulates the secretion of histamine [13]. In addition, decreased TCTP levels have been detected in certain areas of postmortem brains from Down syndrome and Alzheimer’s patients [14]. In previous studies, we demonstrated that TCTP functions as an antiapoptotic protein required for mouse embryonic development and survival in systemic knockout mice [15]. Endoderm markers’, such as Shh and PECAM, expression patterns were markedly reduced in embryonic day 9.5 (E9.5) TCTP^−/−^ embryos, especially in the forebrain and midbrain anterior end. Furthermore, TCTP plays a very modest role in thymocyte development but is critical for peripheral T cell maintenance and TCR-mediated cell proliferation [16]. TCTP is also essential for developmental β-cell mass establishment and adaptation in response to insulin resistance [17]. Recent studies demonstrated that the interacting proteins of TCTP, such as Mcl-1 and Bcl-xL, play roles in the brain. Mcl-1 is a key regulator of apoptosis during the development of the central nervous system (CNS) [18]. Bcl-xL germline-deficient mice also undergo embryonic death at E13 and show increased apoptotic activity in the brain [19]. The conditional disruption of Bcl-xL in catecholaminergic neurons results in viable mice, with the catecholaminergic neuronal population reduced by one-third [20]. These studies suggest that TCTP may play an important role in CNS development. However, despite the importance of TCTP in the regulation of apoptosis, the impact of TCTP disruption during CNS development has not been investigated, except one recent paper that showed that TCTP may be implicated in the regulation of visual axon development of *Xenopus laevis* [21]. Therefore, the neuronal function of TCTP in the brain requires further investigation.

In the present work, we generated and characterized the phenotype of *TCTP* mutant mice and determined the possible mechanisms involved. We showed with a mouse model that TCTP is required for neural development in mammals. Deficiency of TCTP in neuronal and glial cell precursors resulted in decreased bromodeoxyuridine (BrdU) incorporation, increased widespread apoptosis, and disturbance of Tuj1-positive cell maturation, subsequently leading to perinatal death of TCTP mutant mice. Taken together, our results demonstrate that TCTP is required for the survival and differentiation of neuronal progenitor cells and is essential for cortical neurogenesis in development.

## 2. Materials and Methods

### 2.1. Generation of Conditional Knockout Mice, Breeding, and Genotyping

Mice harboring the floxed allele (f) of the *TCTP* gene were generated and genotyped as previously described [15]. Brain neuronal progenitor cell-specific TCTP conditional mutants were obtained by breeding *floxed TCTP* mice with *Nestin-cre* mice (B6.Cg-*Tg (Nes-cre)1Kln*/J, #003771) from Jackson Laboratory [22,23] to produce *Nestin^Cre^*^/+^; *TCTP^flox^*^/+^ (heterozygous, het) mice. The *Nestin^Cre^*^/+^; *TCTP^flox^*^/+^ mice were crossed with *Nestin^Cre^*^/+^; *TCTP^flox^*^/+^ mice or *TCTP^f^*^/*f*^ alone mice to produce *Nestin^Cre^*^/+^; *TCTP^flox^*^/*flox*^ homologous conditional mutant mice (TCTP-cKO). *Nestin^Cre^*^/+^; *TCTP*^+/+^, or *TCTP^f^*^/*f*^ alone mice were used as a control. Both *floxed TCTP* and *Nestin-Cre* mouse lines were generated in C57BL/6 and 129svj mixed background, and the mice used in this study were back-crossed to C57BL/6 for at least 8 generations. Double-heterozygous littermates (*Nestin^Cre^*^/+^; *TCTP^f^*^/+^) were also used as controls for some in vivo experiments. For embryonic time points, the time of plug identification was counted as postnatal day 0.5 (P0.5). Genotyping was performed by PCR using primers P1 (5′-TCTAGAAAAGTGGAGGCGGAGC-3′) and P5 (5′-GGTGACTACTGTGCTTT CGG TA-3′) for the wild-type (450 base pairs) and floxed (520 base pairs) alleles, and cre-sense (5′-TGCCACGACCAAGTGACAGC-3′) and cre-antisense (5′-CCTTAGCGCCGTAAAT CAATCG-3′) for the cre allele (580 base pairs). All animal studies were performed following the recommended procedures approved by the Institutional Animal Care and Use Committee of Tzu Chi University (PPL number: 97043) and conformed to the guidelines of Directive 2010/63/EU of the European Parliament on the protection of animals used for scientific purposes or the National Institutes of Health (NIH) guidelines.

### 2.2. Tissue Processing, Histological Analysis, and Immunohistochemistry

For histological analysis, whole brains were fixed with 4% paraformaldehyde and embedded in paraffin, sectioned, and stained with hematoxylin and eosin. TCTP expression pattern, BrdU incorporation, terminal deoxynucleotidyl transferase-mediated dUTP nick end-labeling (TUNEL) assay, caspase activation, and Oct4 expression were detected by immunohistochemistry with DAB staining. Immunofluorescence was performed by labeling with anti-TCTP (1:200; Abcam, Cambridge, United Kingdom), anti-Tuj1 (1:100; Millipore, Burlington, MA, USA), anti-active caspase-3 (1:100; Abcam), anti-nestin (1:100; Cell Signaling, Danvers, MA, USA), and anti-MAP (1:100; Invitrogen, Carlsbad, CA, USA) antibodies. The retrieved sections were incubated with the primary antibody overnight at 4 °C, washed for 1 h in phosphate-buffered saline (PBS), and incubated with the secondary antibody for 1 h, then washed for 1 h in PBS. Sections were followed by DAB staining, and then counterstained with hematoxylin. For the immunofluorescence sections, visualization of staining was achieved using HiLyte Fluor 488- and HiLyte Fluor 555-conjugated secondary antibodies (1:200; AnaSpec, Fremont, CA, USA), then counterstained with 4’, 6-diamidino-2-phenylindole (DAPI) for double or triple immunofluorescence staining. Immunofluorescence was visualized and captured using an Olympus BX43 upright fluorescent microscope equipped with a digital camera system (DP-72, Olympus, Tokyo, Japan) or a Zeiss LSM 510 META confocal imaging system (Carl Zeiss, Oberkochen, Germany).

### 2.3. RNA Isolation, cDNA Synthesis, and Quantitative Real-Time Reverse Transcriptase PCR

To analyze gene expression in the embryonic and postnatal stages of control and TCTP-cKO mice, RNA was isolated from 4 independent biological samples at stages E13.5, E16.5, P0.5, and P56 using TRIzol reagent (Invitrogen #15596018). Total RNA (1 μg) extracted from brain tissue was reverse-transcribed to synthesize cDNA using a ReverTra Ace-α- reverse transcription kit (Toyobo #F0937K, Toyobo, Osaka Prefecture, Japan) and oligo (dT) as a primer. Real-time PCR was performed using Power SYBR Green PCR Master Mix (Applied Biosystems #4368577, Applied Biosystems, Foster City, CA, USA) in an ABI 7300 Real-Time PCR System (Applied Biosystems). The specific primers used for the detection of genes are shown in Table 1. Each reaction was performed in duplicate, and dissociation curves were constructed to ensure that only a single product was amplified. The transcript expression level of the gene of interest was normalized to GAPDH mRNA as the internal control, and the data were expressed as a fold change over the control group.

### 2.4. Cell Proliferation Assay

Bromodeoxyuridine (5-bromo-2-deoxyuridine, BrdU) is a synthetic nucleoside that is an analogue of thymidine. BrdU can be incorporated into the newly synthesized DNA of replicating cells (during the S phase of the cell cycle), substituting for thymidine during DNA replication. For the labeling of cells in the S phase, pregnant mice (E13.5, E16.5, E17.5) obtained from timed mating were injected intraperitoneally with 10 mg BrdU (Invitrogen #000103) per 100 g of body weight. The brains dissected from E13.5, E16.5, and neonatal offspring (P0.5) were recovered in ice-cold PBS at pH 7.4 and fixed in 4% paraformaldehyde. Incorporation of modified nucleotide was detected by staining with anti-BrdU antibody (BU-20) as described previously [24].

### 2.5. TUNEL Assay

Cells in embryos undergoing apoptosis were analyzed by TUNEL assay using the In Situ Cell Death Detection Kit (Roche Diagnostics, Basel, Switzerland) according to the manufacturer’s instructions. After TUNEL staining, the sections of brain were counterstained using hematoxylin.

### 2.6. Primary Neuronal Cultures

Primary cortical culture was prepared from fetal cortices of *Nestin^Cre^*^/+^; *TCTP^f^*^/+^ intercrossed with *TCTP^f^*^/*f*^ mice at embryonic day 16.5 (E16.5) or postnatal day 0.5 as previously described [25]. Briefly, the fetal cortices were removed and dissected, followed by mechanical trituration in Hanks’ balanced salt solution (GIBCO #14185, Thermo Fisher, Waltham, MA, USA) containing 2.5 U/mL dispase and 2 U/mL DNase. The supernatant that contained cortical neurons was filtrated through a 70-μm filter (BD Falcon #REF352350, New York, NY, USA) and transferred into a 15-mL autoclaved tube, and then immediately centrifuged at 1500× *g* for 10 min. The pellet containing neurons was resuspended in minimum essential medium (MEM) (GIBCO #12561) containing 10% heat-inactivated fetal bovine serum (FBS), 10 g/L glucose (Sigma #G7021, St. Louis, MI, USA), 0.176 g/L L-glutamine (GIBCO #25030), 0.12 g/L sodium pyruvate (Sigma #p2256), 2.2 g/L sodium bicarbonate, 0.238 g/L HEPES (Sigma #H4034), and 10 mL/L 100× penicillin–streptomycin (BioWest #L0022, Les Ulis, France). Cells were seeded at a density of 2.5 × 10^5^/well in 0.5 mL medium in a 24-well culture plate. The culture dishes were precoated with poly-d-lysine hydrobromide (50 μg/mL) (BD Bioscience #354210) for 2 h. The dishes were then washed twice with autoclaved deionized water. After 4 h, the MEM was replaced by Neurobasal medium (GIBCO #21103-049) supplemented with B27 (GIBCO #17504-044). Cells were incubated at 37 °C in a humidified atmosphere of 5% CO_2_ and 95% air.

### 2.7. Cortical Progenitor Cultures and Immunofluorescence

Cortical progenitor cells were cultured as described previously [26]. Briefly, cortices were dissected from TCTP-cKO and littermate control embryos at E14.5–E15.5. Cortices were mechanically dissociated by trituration, and cell aggregates were plated on polyornithine-coated 4-well dishes and cultured in media containing Neurobasal medium (Invitrogen), 0.5 mM glutamine, 0.5 % penicillin–streptomycin, 1% N2 supplement (Invitrogen), 2% B27 supplement (Invitrogen), and 10 ng/mL NGF-2. On day 1, the medium was replaced with fresh medium. Immunofluorescence or immunohistochemistry experiments were performed 3 days after culture. Cultured cells were fixed in 4% paraformaldehyde for 20 min at room temperature and further processed for immunostaining. Cells were permeabilized with 0.1% Triton X-100, blocked for 1 h in 5% bovine serum albumin–5% goat serum, and incubated with primary antibodies, rabbit TCTP, and anti-nestin. After incubation overnight, cells were washed with PBS followed by 2 h of incubation with secondary antibodies, conjugated FITC, or rhodamine. Cells were counterstained for 30 s with DAPI for double immunofluorescence.

### 2.8. Cell Survival Assay and MTT Reduction Assay

Quantitative measurements of cortical progenitor cell survival were performed using the 3-[4, 5-dimethylthiazol-2-yl]-2, 5-duphenyltetrazolium bromide (MTT) reduction assay. The MTT reduction assay measures mitochondrial function as an index for cell survival. Cells were incubated in culture medium with MTT at a final concentration of 0.5 mg/mL MTT for 2 h; afterwards, the culture medium was replaced with 500 mL of dimethyl sulfoxide. Absorbance at 570 nm was determined by an ELISA reader.

### 2.9. Statistical Analysis of the Data

The experimental results are expressed as the mean and standard error of the mean (mean ± SEM) accompanied by the number of observations. Data were analyzed by Student’s unpaired *t*-test, taking *p*-values of <0.05 as significant. Data obtained from 3 or more groups were compared by one-way ANOVA; *p*-values of <0.05 were considered statistically significant. Because the sample sizes involved in each experiment were different, they were included in the figures and/or figure legends.

## 3. Results

### 3.1. Expression Pattern of TCTP in the Mouse Brain during Development

**Figure 1:**
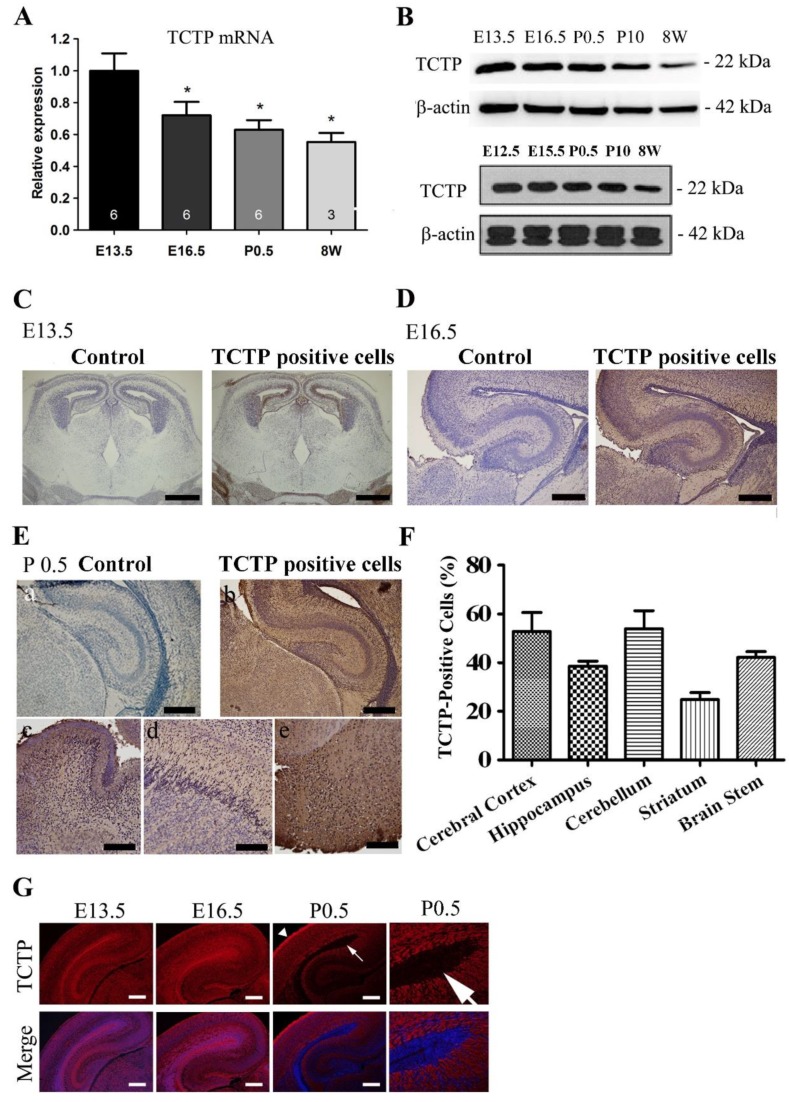
Expression pattern of translationally controlled tumor-associated protein (TCTP) from the embryonic to adult stage in the brain. (**A**) Relative TCTP mRNA expression at different stages in the whole brain from wild-type mice was estimated by qRT-PCR (data presented as mean ± SEM, * *p* < 0.05 compared with embryonic day 13.5 (E13.5), one-way ANOVA, *n* = 3–6 per group). (**B**) TCTP protein in the whole brain from control mice (*TCTP^flox^*^/*flox*^) at E12.5, 13.5, E15.5, 16.5, and postnatal day 0.5, 10, and 56 was estimated by Western blotting. (**C**–**E**) TCTP protein expression pattern in whole-brain sagittal sections from wild-type mice at E13.5 and coronal sections at E16.5 and P0.5, including the (a) cerebral cortex, (b) hippocampus, (c) cerebellum, (d) striatum, and (e) brain stem, detected by immunohistochemistry with DAB staining (brown stain indicates TCTP-positive cells) (*n* = 3 for each time point). (**F**) Summarized quantification of TCTP protein expression in (**E**). (**G**) Representative TCTP (red) and 4’,6-diamidino-2-phenylindole (DAPI)/TCTP double immunofluorescence staining of brain sections from C57BL/6 mice at E13.5, E16.5, and P0.5; *n* = 3. The arrow indicates the ventricular zone area; the arrowhead indicates the cortical layer. Scale bars: C, 600 μm; D, 200 μm; E: a, c, d, e, 100 μm, b, 200 μm; G, 200 μm.

We demonstrated that TCTP plays a critical role in survival at the mid-embryonic stage, but its role and functional mechanism in the regulation of neurogenesis at the early stage of brain development remains unknown. To address this question, we first examined whether TCTP is expressed in the developing brain and other populations of cells. TCTP RNA expression can be detected in the neural ectoderm and nervous system in mouse embryos at the E10.5 stage with in situ hybridization (Supplementary Figure S1), and the whole brain in E13.5, E16.5, P0.5, and P56 mice with qPCR (Figure 1A) with specific primers (Table 1), suggesting the presence of TCTP RNA from E13.5 to P10 involved in neural development. To gain insight into the TCTP function during brain development, we analyzed TCTP protein expression profiles in the CNS during development. As shown in Figure 1B, immunoblotting with a specific anti-TCTP antibody revealed that TCTP protein was highly expressed in whole brains from E13.5, E16.5, P0.5, and P10 but not mature mice (P56), as shown in the upper panel. The lower panel shows a similar TCTP protein expression pattern from E12.5 to adult. A widespread TCTP protein expression pattern was confirmed by immunohistochemistry analysis in whole-brain sections from mice at E13.5 (Figure 1C), E16.5 (Figure 1D), and postnatal day 0.5 (Figure 1E), including the striatum, olfactory bulb, cerebral cortex, hippocampus, cerebellum, and brain stem. The quantification data are summarized in Figure 1F. Furthermore, TCTP protein at E13.5 detected by double immunofluorescence staining was highly expressed in the ventricular zone (Figure 1G), where neural progenitor cells are concentrated. We found that TCTP expression in the ventricular zone was dramatically decreased to a low level from E16.5 to P0.5 but increased in the cortical layer. The abundant expression pattern of TCTP in the brain suggests that it may play an important role in CNS development. To further investigate the role of TCTP in nervous system development, we disrupted TCTP expression with conditional knockout mice, specifically in the neural progenitor cells of the brain.

### 3.2. Generation of Conditional TCTP Knockout Mice

**Figure 2:**
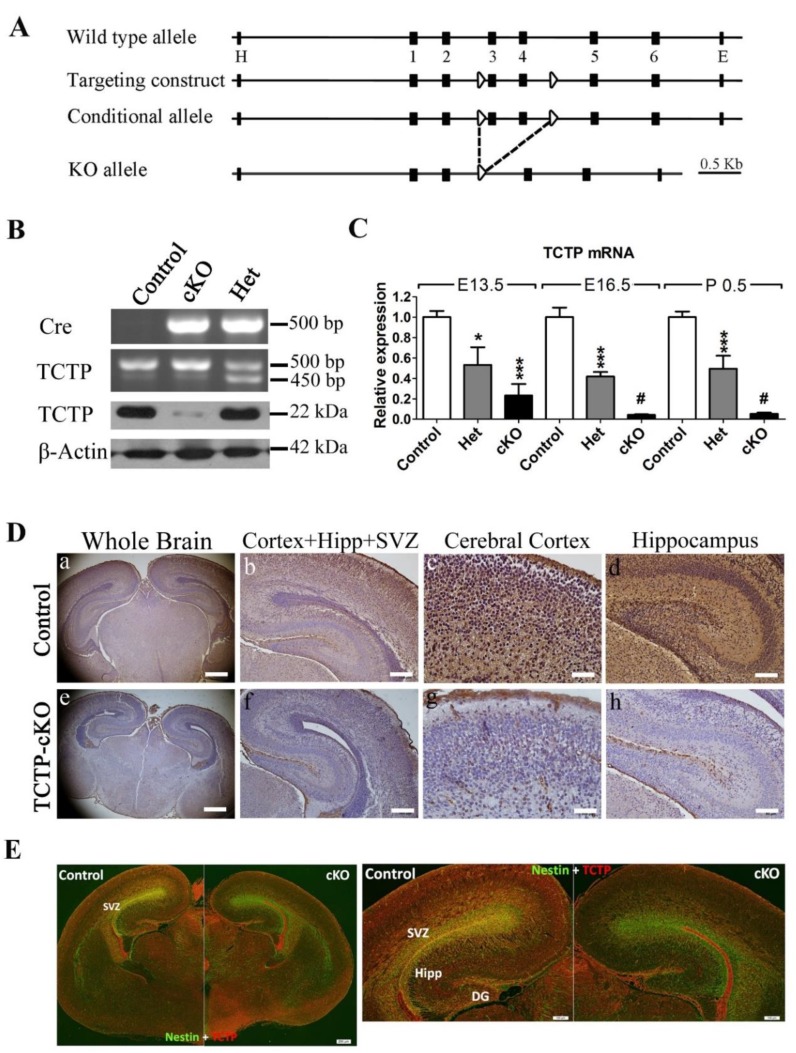
Generation of conditional TCTP knockout mouse model. (**A**) Diagram of the targeting strategy for the generation of the conditional TCTP allele. The targeting construct contains *loxP* sites (arrowheads) flanking exons 3–4 and restriction sites EcoRI (E) and HindIII (H). (**B**) Genotyping with PCR, top panel, and Western blotting of TCTP expression, bottom panel, from *Nestin*^+/+^; *TCTP^flox^*^/*flox*^ (Control), *Nestin^Cre^*^/+^; *TCTP^flox^*^/*flox*^ (heterozygous), and N*estin^Cre^*^/+^; *TCTP^flox^*^/+^ (cKO) mice at P0.5. (**C**) qRT-PCR of relative TCTP mRNA expression in the whole brain from control, heterozygous, and conditional knockout mice at different stages was estimated (data presented as mean ± SEM, * *p* < 0.05, *** *p* < 0.001 compared with control, *#* < 0.05 compared with E13.5 cKO, one-way ANOVA, *n* = 3–11 per group). (**D**) Immunohistochemistry of TCTP protein from control and TCTP-cKO mice at P0.5. DG, dentate gyrus; Hipp, hippocampus; SVZ, subventricular zone. Scale bars: a, e, 400 μm; b, f, 200 μm; c, g, 40 μm; d, h, 100 μm. (**E**) Sections at E16.5 were also detected by double-labeled immunofluorescence with antibodies against TCTP to identify the deletion and Nestin to identify neural precursor cells. A representative image is shown for three independent experiments. Scale bars: 100 μm for right panel, 200 μm for left panel.

To circumvent the embryonic lethality of complete disruption of the *TCTP* gene [15] and to examine the role of TCTP in CNS development, we used the Cre/loxP gene targeting system to generate neuronal progenitor cell-specific TCTP conditional mutants, which were obtained by breeding *TCTP^f^*^/*f*^ mice with *Nestin-cre* transgenic mice from Jackson Lab [22,23] to produce TCTP-cKO mice (Figure 2A). The *Nestin-Cre* transgenic mice expressed Cre recombinase under the control of the rat *Nestin* promoter. The *Nestin-cre*-mediated recombination was first present in precursor cells of neurons and glia around embryonic day 11 and was detected in the cortical wall, cortical layers, ventricular zone (VZ), subventricular zone (SVZ), and intermediate zone (IZ) of the telencephalon and spinal cord of the growing population of postmitotic neurons in the developing central nervous system [27]. The generation and characterization studies of TCTP-cKO conditional mutants could help us to selectively assess the role of TCTP in neural precursor cells during central nervous system development.

Genotype analysis of progeny at P10 from *Nestin^C^^re^*^/+^; *TCTP^f^*^/+^ mice crossed with *TCTP^f^*^/*f*^ revealed that no homozygous offspring were found, suggesting embryonic or neonatal death of TCTP-cKO mice. Therefore, we checked the embryo variability for TCTP knockout mice. Homozygous offspring could be found at E9.5, E10.5, E13.5, E14.5, and E16.5. The TCTP disruption genotype *Nestin^C^^re^*^/+^; *TCTP^f^*^/*f*^ was confirmed by PCR in newborn mice, postnatal day 0.5 (Figure 2B). The four offspring genotypes (*Nestin*^+/+^; *TCTP^f^*^/*f*^, *Nestin*^+/+^; *TCTP^f^*^/+^, *Nestin^C^^re^*^/+^; *TCTP^f^*^/+^, *Nestin^C^^re^*^/+^; *TCTP^f^*^/*f*^) were identified in an expected Mendelian ratio (1:1:1:1). The *Nestin-cre*-derived deletion of TCTP was further demonstrated by TCTP mRNA level with qRT-PCR (Figure 2C), and protein expression with immunoblotting (Figure 2B, lower panel) and immunohistochemistry (Figure 2D). The sections at E16.5 were also detected by immunofluorescence double labeled with antibodies to TCTP to identify the *TCTP* deletion and antibodies against *Nestin* to identify neural precursor cells (Figure 2E). Mice heterozygous for the deleted allele of *TCTP* (*Nestin^C^^re^*^/+^; *TCTP^f^*^/+^) were viable, fertile, and not morphologically different from wild-type (*Nestin^C^^re^*^/+^; *TCTP*^+/+^) littermates.

### 3.3. Loss of TCTP in Neuronal Progenitor Cells Resulted in Early Neonatal Death

**Figure 3:**
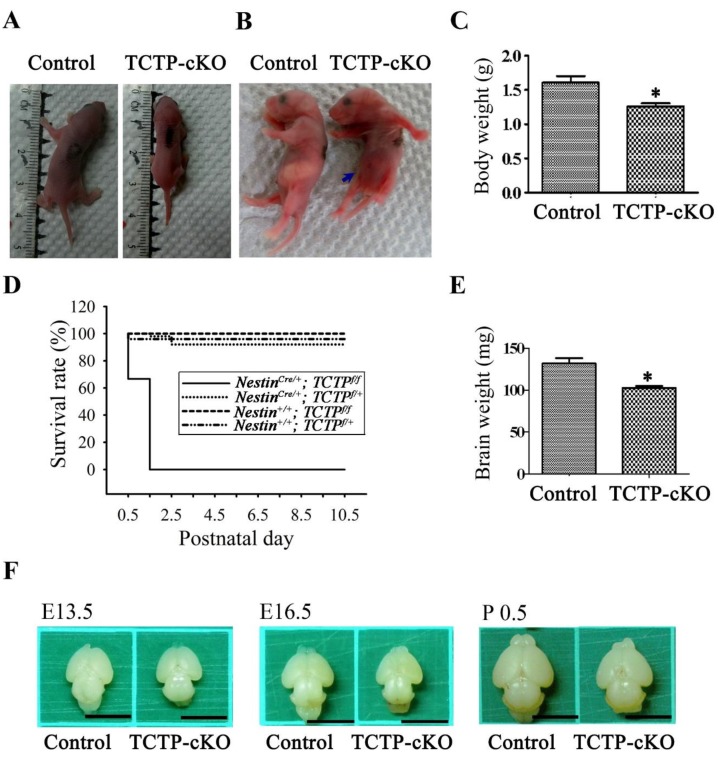
Phenotypes and survival rates of TCTP-cKO mice. (**A**) Body size of control and TCTP-cKO mice at P0.5. (**B**) Milk in stomachs of control and cKO mice at P0.5. (**C**) Body weight of control and cKO mice at P0.5 (data presented as mean ± SEM, * *p* < 0.05, Student’s *t* test, *n* = 3 per group). (**D**) Survival rate of offspring from *Nestin^C^^re^*^/+^; *TCTP^f^*^/+^ mice crossed with *TCTP^f^*^/*f*^ mice during development from P0.5 to P10.5. (**E**) Brain weight of control and cKO mice at P0.5 (data presented as mean ± SEM, * *p* < 0.05, Student’s *t* test, *n* = 4 per group). (**F**) Morphology of brains from control and cKO mice at E13.5, E16.5, and P0.5. Scale bar: F, 0.5 cm.

Compared with littermate controls, all the surviving TCTP-cKO mutant pups at P0.5 were smaller in body size (Figure 3A) and did not suckle well (Figure 3B, indicated by arrow), as demonstrated by the lack of milk in their stomachs. Abnormal behavior, particularly ataxia, was observed in cKO pups. Moreover, body weight, brain size, and brain weight were reduced in TCTP mutant pups examined at P0.5 when compared with littermate controls (Figure 3C,E,F). We found that the TCTP mutant mice exhibited early postnatal lethality phenotype at P1.5 to P2.5 (Figure 3D, Table 1). Mice heterozygous for the deleted allele of *TCTP* (*Nestin^C^^re^*^/+^; *TCTP^f^*^/+^) were viable, fertile, and not morphologically different from wild-type (*Nestin^C^^re^*^/+^; *TCTP*^+/+^) littermates. The perinatal death of TCTP-cKO mice might be caused by abnormal CNS development and malnutrition from a lack of milk in the stomach.

### 3.4. TCTP is Required for Cortical Neurogenesis

**Figure 4:**
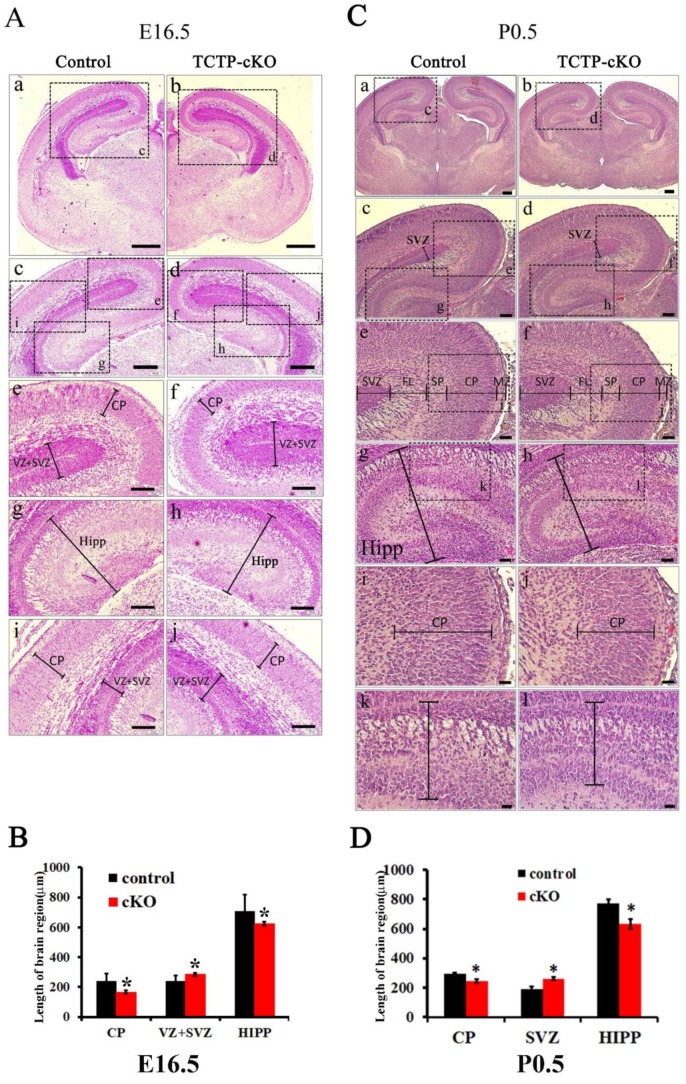
Histological analysis of E16.5 embryos and P0.5 pups from control and cKO mice. (**A**) Control (*Nestin^Cre^*^/+^; *TCTP^w^*^/*w*^; a, c, e, g, and i) and cKO (*Nestin^Cre^*^/+^; *TCTP^f^*^/*f*^; b, d, f, h, and j) brain sections from E16.5 embryos were stained with hematoxylin and eosin. (**B**) Bar graph summarizing quantification of the sizes of brain regions in (**A**). (**C**) Control (a, c, e, g, i, and k) and cKO (b, d, f, h, j, and l) brain sections from P0.5 pups were stained with hematoxylin and eosin. (**D**) Bar graph summarizing the quantification of the sizes of brain regions in (**C**), (data presented as mean ± SEM, * *p* < 0.05, Student’s *t* test, *n* = 3 per group). A representative image is shown for three independent experiments. MZ, marginal zone; SP, subplate. Scale bars on panel A: a, b, 400 μm; c, d, 200 μm; e–j, 100 μm. Scale bars on panel B: a, b, 400 μm; c, d, 200 μm; e–h, 100 μm; i–l, 40 μm.

The perinatal death of TCTP-cKO mice prompted us to further detect the brain morphology of cKO mice compared with littermate controls by hematoxylin and eosin Y staining. To assess the role of TCTP in brain development, brains were collected at E16.5, around mid-neurogenesis, and P0.5. This stage is characterized by a large population of Nestin-expressing progenitors within the VZ and subventricular zone (SVZ), early committed neuroblasts within the SVZ/intermediate zone (IZ), and a growing population of postmitotic neurons in the developing cortical plate (CP). Therefore, we examined the cortical plate, hippocampus, and lamination of SVZ in the cerebral cortex in TCTP-deficient brains compared with littermates (Figure 4A,B). The cortical plate and hippocampus region of the TCTP-cKO mouse brain was significantly decreased as expected. In contrast, the VZ/SVZ region in the cKO mouse brain was significantly increased. Enlargement of the VZ/SVZ region in TCTP-conditional null mice indicated that perhaps some progenitor cells cannot fully migrate and commit to differentiation or are unable to exit the cell cycle. TCTP-cKO mice at P0.5 also exhibited a size reduction in the cortical plate and hippocampus but broad and lower levels of staining in the proliferative SVZ and VZ compared with littermate controls (Figure 4C,D). The structural abnormality in the mutant brain at E16.5 and P0.5 suggests that the selective deletion of TCTP in neural progenitor cells significantly impaired the development of the cerebral cortex surrounding the lateral ventricle (Figure 4).

**Figure 5:**
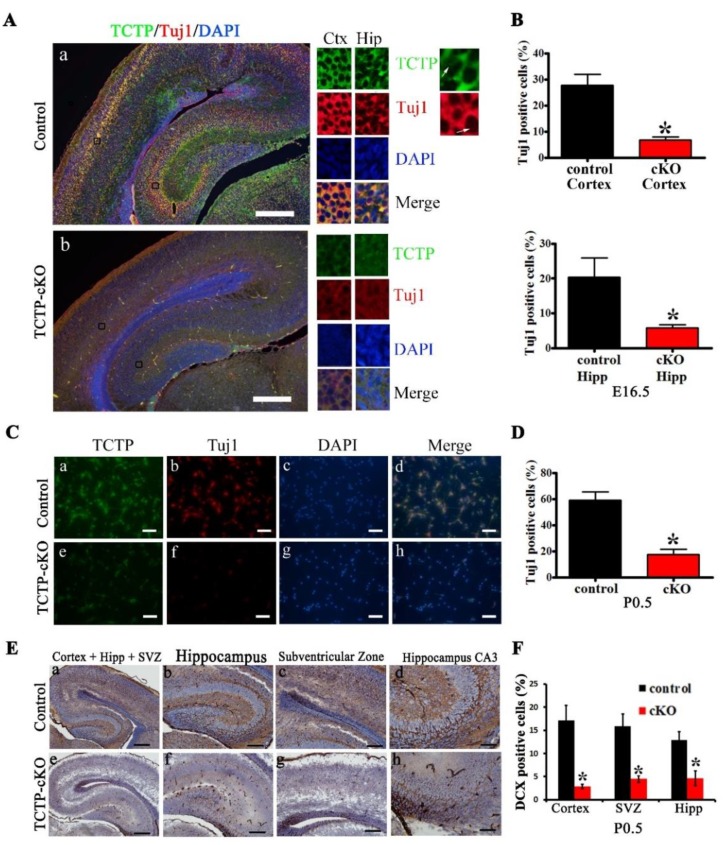
Central nervous system (CNS) neurogenesis of TCTP-cKO and control mice was detected. (**A**) Double immunofluorescence staining detected TCTP (green) and Tuj1 (red) of brain sections at E16.5. Bottom panel shows magnification of panels a and b. (**B**) Bar graph summarizing the quantification of Tuj1-positive cells in (**A**). (**C**) TCTP (green) and Tuj1 (red) of primary cultured cortical neurons from control and TCTP-cKO mice at P0.5 were detected. (**D**) Bar graph summarizing the quantification of Tuj1-positive cells in (**C**). (**E**) The neurogenesis marker doublecortin (DCX) was detected in TCTP mutant brains from control and TCTP-cKO mice at P0.5. (**F**) Bar graph summarizing the quantification of DCX-positive cells in (**E**). (Data presented as mean ± SEM, * *p* < 0.05, Student’s *t* test, *n* = 3 per group.) Scale bars: A, 200 μm, B, 40 μm, C, a, e, 200 μm; b–d, f–h, 100 μm. A representative picture is shown for three independent experiments.

Furthermore, Tuj1 was expressed not only in newly committed immature postmitotic neurons but also in differentiated neurons and in some mitotically active neuronal precursors [28]. Therefore, the brain sections and isolated primary cortical neurons from control and TCTP conditional knockout mice at E16.5 or P0.5 were prepared for detection by double immunofluorescence staining with antibody against TCTP and Tuj1. We found that the expression of Tuj1 was reduced in the cortex and hippocampus of the brain sections (Figure 5A,B) and primary cortical neurons (Figure 5C,D) from TCTP-cKO mice at E16.5 and P0.5 by immunofluorescence analysis. The results exhibited decreased neurogenesis in the TCTP cKO mice compared with control mice (Figure 5A–D). To further investigate the impact of TCTP deletion on neurogenesis, we examined several cell-type-specific markers in the cerebral cortex, hippocampus, VZ, and SVZ. Nestin protein is expressed at high levels in cortical radial glia/neural progenitor cells (NPCs) [29]. In contrast, doublecortin (DCX) expression is low in NPCs but upregulated in postmitotic neurons [30]. Nestin and DCX are thus routinely used as markers to distinguish these mutually exclusive cell types. DCX, a marker for newly migrating populations, could affect neuronal migration by regulating the organization and stability of microtubules [31]. Compared to littermate controls, the brains of cKO mutant mice exhibited a significant reduction of neurogenesis marker DCX-positive neurons (Figure 5E,F) by immunohistochemistry analysis. Moreover, an in vitro study of primary cell culture indicated that high expression of TCTP was observed in both proliferating neural precursors (Nestin+) and mature neurons (MAP2+) during brain development. Some TCTP-negative cells from mutant mice expressed Nestin and survived at first (Supplementary Figure S2A). In contrast, most surviving cells cultured from mutant mice expressed TCTP and MAP but not TCTP-negative cells (Supplementary Figure S2B,C). These results indicate that TCTP is involved in neurogenesis in the embryonic and early postnatal stages. Specifically, *Nestin-cre*-derived TCTP disruption resulted in decreased Tuj1-positive newly committed neurons, DCX, and Nestin-positive neural progenitors, indicating that TCTP is required for the survival of these cell populations. Furthermore, we also demonstrated that a deficiency of TCTP possibly delayed or impaired neuronal proliferation and cell cycle progression.

### 3.5. Decreased Cell Proliferation in Nestin-Cre-Derived TCTP-Deficient Mouse Brain

**Figure 6:**
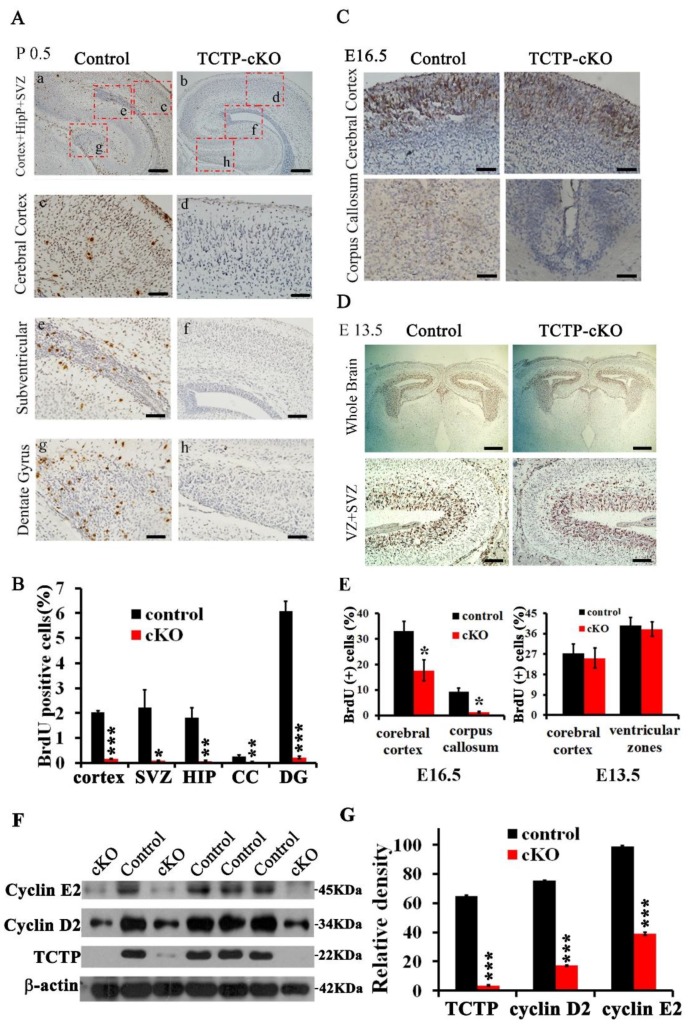
Cell proliferation and cyclin protein expression detected in brain sections and protein lysates from control and cKO mice. (**A**) Bromodeoxyuridine (BrdU) incorporation assay of control and TCTP-cKO mice was estimated at P0.5. (**B**) Bar graph summarizing the quantification of BrdU-positive (+) cells in (**A**). (**C**,**D**) BrdU incorporation in brain sections from control and TCTP-cKO mice was also detected at E16.5 and E13.5 (**E**) Bar graph summarizing the quantification of BrdU-positive cells in (**C**,**D**). (Values are mean ± SEM, Student’s *t* test, * *p* < 0.05 compared with control group; *n* = 3). CC. corpus callosum; DG, dentate gyrus; HIP, hippocampus; SVZ, subventricular zone. Scale bars: A: a, b, 200 μm; c, d, e, g, h, 40 μm; f, 100 μm. C: 40 μm; D: top panel, 400 μm; bottom panel, 40 μm. (**F**) Western blotting of cyclins D2 and E2 and TCTP in control and TCTP-cKO mice at P0.5. (**G**) Bar graph summarizing quantification of Western blotting relative density in (**E**). Relative density is presented as the ratio of band intensity of target protein to internal control-actin. Values are mean ± SEM. Data were statistically analyzed by student’s *t* test; * *p* < 0.05, ** *p* < 0.01, *** *p* < 0.001 compared with control group; *n* = 3).

We next examined whether the cellular proliferation and apoptosis involved the phenotype of the TCTP-cKO mutant mice. To address this issue, BrdU incorporation and TUNEL assays were determined. We examined whether loss of TCTP causes the defect in brain development and neuronal cell proliferation. The proportion of cells in the S phase of the cell cycle was determined by immunohistochemistry. High levels of BrdU expression were detected in the hippocampus, dentate gyrus, marginal zone, and subventricular zone of the cerebral cortex in the littermate control mice at P0.5; however, BrdU-positive cells were reduced in the TCTP mutant mice (Figure 6A). The quantification data at P0.5 are summarized in Figure 6B. A significant reduction of BrdU incorporation was also found in the cKO mice at E16.5 (Figure 6C), especially in the corpus callosum, but not at E13.5 (Figure 6D). The quantification data at E16.5 and E13.5 are summarized in Figure 6E. These results are consistent with the *Nestin-cre* activity, which was high in the preplate and low in the ventricular zone (VZ) at E12.5, and high in the cortical plate (CP) and intermediate zone (IZ) and low in the VZ and SVZ at E14.5. Then, the scope of recombination expanded to neural stem cells (NSCs) and NPCs increased dramatically during E14.5 to E17.5 [32]. To determine whether decreased proliferation was correlated with perturbation of cell cycle progression, immunoblotting analysis was carried out to compare the expression levels of cyclins D2 and E2 between the *Nestin^Cre^*^/+^; *TCTP^f^*^/*f*^ pups and littermate controls. We found that cyclin D2 and E2 expression was significantly suppressed in the TCTP-deficient brain (Figure 6F,G), which may delay or impair cell cycle progression and neuronal proliferation. These results were consistent with the BrdU incorporation assay and DCX immunohistochemistry staining. The reduced levels of cyclins D2 and E2 in *Nestin^Cre^*^/+^; *TCTP^f^*^/*f*^ pups was also consistent with what we demonstrated: That mice with systemic disruption of TCTP exhibited decreased expression of cyclin D2 and E2 in E9.5 TCTP^−/−^ embryos [15]. These results indicate that TCTP is required for cortical neurogenesis.

### 3.6. Disruption of TCTP-Induced Cell Apoptosis and Cell Autonomous Behavior

**Figure 7:**
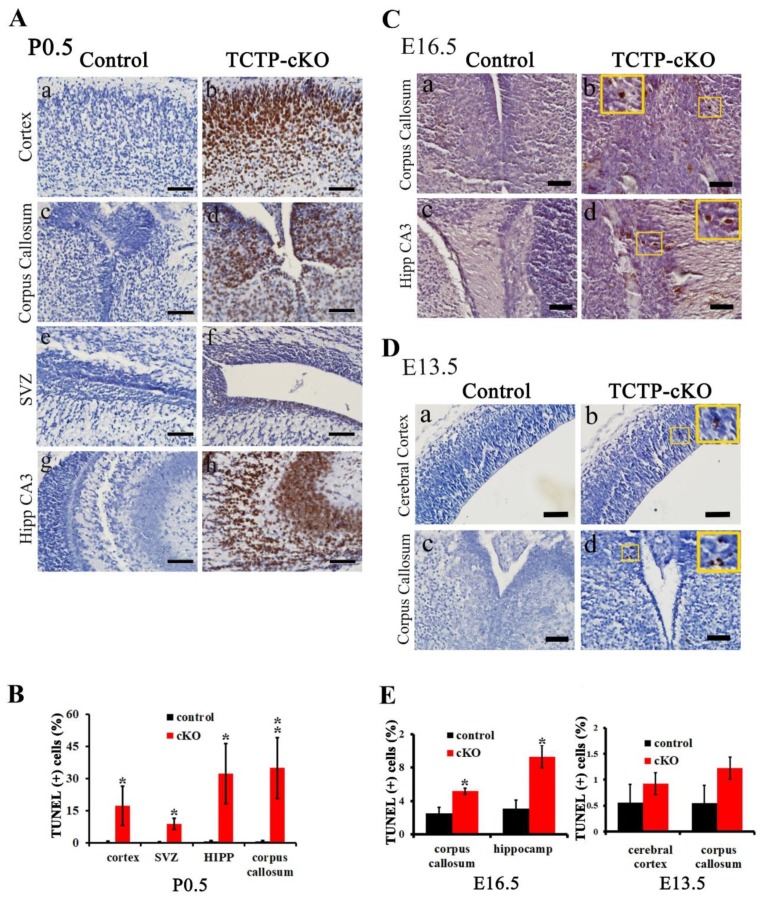
Cell apoptosis was detected in brain sections from control and cKO mice. (**A**) TUNEL staining was performed on coronal sections of the brain at P0.5, including the (a,b) hippocampus, (c,d) corpus callosum, (e,f) subventricular zone, and (g,h) hippocampus CA3. (**B**) Bar graph summarizing the quantification of TUNEL-positive (+) cells in (**A**). (Values are mean ± SEM, Student’s *t* test, * *p* < 0.05, ** *p* < 0.01 compared with control group; *n* = 3). (**C**,**D**) Apoptosis of brain sections from TCTP-cKO and control mice at E16.5 (*n* = 3) and E13.5 (*n* = 4) was also detected by TUNEL staining assay. Boxes indicate magnification. (**E**) Bar graph summarizing quantification of TUNEL-positive cells in (**C**,**D**). (Values are mean ± SEM, Student’s *t* test, * *p* < 0.05 compared with control group). Hipp, hippocampus; SVZ, subventricular zone. Scale bar: A, C, 40 μm; D, 100 μm.

Next, we determined whether cell-specific disruption of TCTP expression leading to increased apoptotic cell death may contribute to the decrease in different kinds of neurons and neonatal death. To address this hypothesis, TUNEL assay was conducted at postnatal day 0.5. TUNEL staining was performed on coronal sections of the brain, including the hippocampus and corpus callosum. Increased TUNEL-positive cells (brown staining) were detected in *Nestin-cre*-derived TCTP-deficient brain compared with littermate controls (Figure 7A). The apoptotic cells were significantly increased in the cerebral cortex, subventricular zone, hippocampus, and corpus callosum in TCTP-cKO mutant compared with controls at P0.5 (Figure 7A,B) and E16.5 (Figure 7C) but not E13.5 (Figure 7D). The quantification data at E16.5 and E13.5 are summarized in Figure 7E.

**Figure 8:**
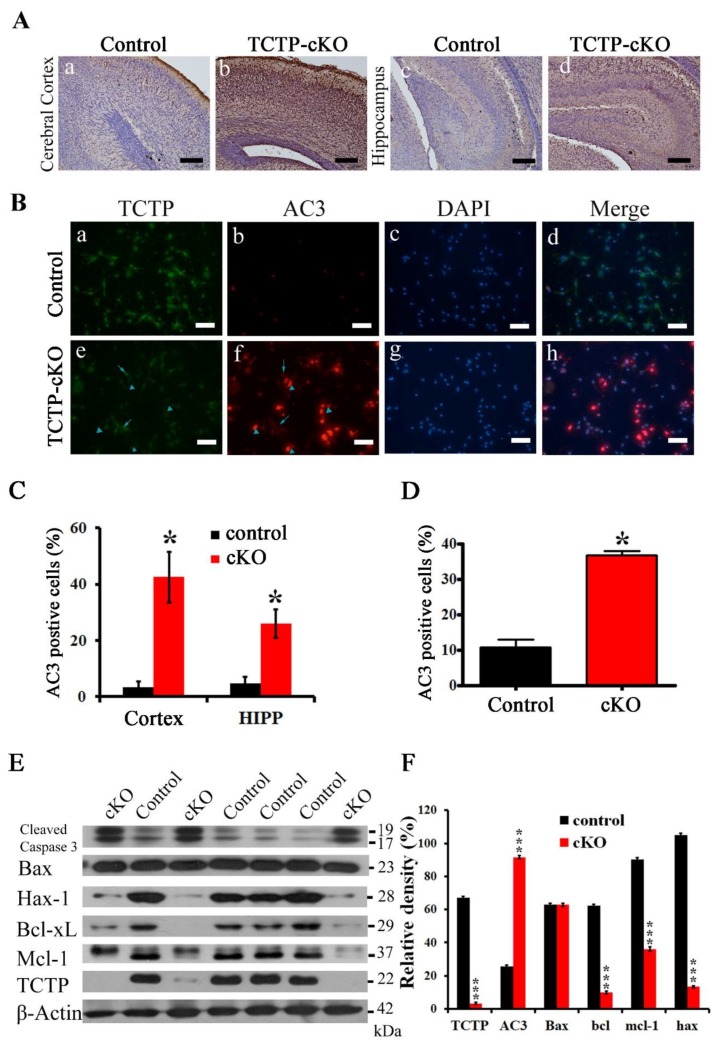
Caspase cleavage apoptosis was detected by immunohistochemistry, immunofluorescence, and Western blot. Apoptotic and antiapoptotic related protein levels were detected by Western blot. (**A**) Cerebral cortex and hippocampus of control and TCTP-cKO mice were stained for active caspase-3 (AC3) by immunohistochemistry at P0.5. (**B**) Double immunofluorescence staining for TCTP (green) and AC3 (red) of primary neuronal cell from control and TCTP-cKO mice at E16.5. The arrow indicates TCTP-positive cells, and the arrowhead indicates TCTP-negative cells. (**C**) Bar graph summarizing the quantification of active caspase-3 staining in (**A**). (**D**) Bar graph summarizing the quantification of active caspase-3 staining in (**B**). (Data presented as mean ± SEM, * *p ≤* 0.05, Student’s *t* test, *n* = 3 per group). (**E**) Protein levels of cleaved caspase-3, bax, hax-1, bcl-xl, mcl-1, and TCTP in control and TCTP-cKO mice were measured by Western blotting at P0.5. (**F**) Bar graph summarizing quantification of Western blot in (**E**). Relative density is presented as the ratio of band intensity of target protein to the internal control, β-actin. (Data presented as mean ± SEM, *** *p <* 0.001, Student’s *t* test, *n* = 3 per group). Scale bars: A, 100 μm; B, 40 μm.

We further evaluated whether the observed neuronal cell loss surrounding the mouse lateral ventricle resulted from apoptosis with another apoptosis marker. Coronal sections at P0.5 from *Nestin-cre*-mediated TCTP mutant brains and from control littermates were detected by immunohistochemistry with antibody agonist active caspase 3 (AC3), a hallmark of classical apoptotic cell death [18]. Numerous active caspase 3-positive cells were present throughout the cortical plate, VZ/SVZ, CP, and hippocampus of TCTP mutant mice, whereas only an occasional apoptotic cell was observed in littermate controls (Figure 8A,C). These data were consistent with the decrease of Tuj1- and DCX-positive neurons (Figure 5A,C,E) in mutant mice, suggesting that the increased apoptosis also involved the phenotype of the TCTP-cKO mutant mice. Immunohistochemistry with primary neuronal cultures was used to clarify whether the cell death of TCTP conditional knockout mice was cell autonomous or nonautonomous. The primary neuronal cultures from the TCTP-cKO mutant mice at E16.5 showed enhanced apoptotic cell death compared with control mice (Figure 8B,D). TCTP-negative cells (arrowhead), but not TCTP-positive cells (arrow), exhibited active caspase-3 positive signal (Figure 8B). This massive cell death by apoptosis observed in brain sections in vivo and TCTP-negative cells in vitro indicated that the cell death from *Nestin-cre*-derived TCTP disruption specifically in neuronal progenitor cells was caused by a cell autonomous mechanism. Again, TCTP is required for cortical neurogenesis.

Apoptotic cell death in the brain of TCTP-cKO mutant mice was also confirmed with the increased cleaved caspase-3 by Western blot. Among the interacting proteins of TCTP, we first investigated the expression of mMcl-1 [9] and Bcl-xL [10], both antiapoptotic members of the Bcl-2 family. TCTP protects from apoptotic cell death by antagonizing bax function in cooperation with mMcl-1 and Bcl-xL [33]. The *Nestin-cre*-driven TCTP knockout mice exhibited decreased expression levels of mMcl-1 and Bcl-xL but not bax protein (Figure 8E,F) compared with littermate controls. The disruption of TCTP caused Bcl-xL protein degradation (Figure 8E,F) but not decreased DNA transcription.

Antiapoptotic protein hax-1 was also reduced in mutant pups. These results demonstrate that TCTP plays a critical role in the developing nervous system when using TCTP-cKO mice. The increased apoptosis and decreased proliferation may contribute to early neonatal death. The phenotype of increased neuronal loss through apoptotic cell death in the brain of TCTP-cKO knockout mice might be attributed to the decreased cyclin D2, Mcl-1, Bcl-xL, and hax-1 expression. Therefore, TCTP is essential for the maintenance of neural precursor cell survival and differentiation during CNS development.

### 3.7. Conditional Deletion of TCTP in Neuron Progenitor Cells Resulted in Decreased Cell Survival and Suppression of Transcription Factor Oct4 Expression in Cortical Progenitor Cultures

**Figure 9:**
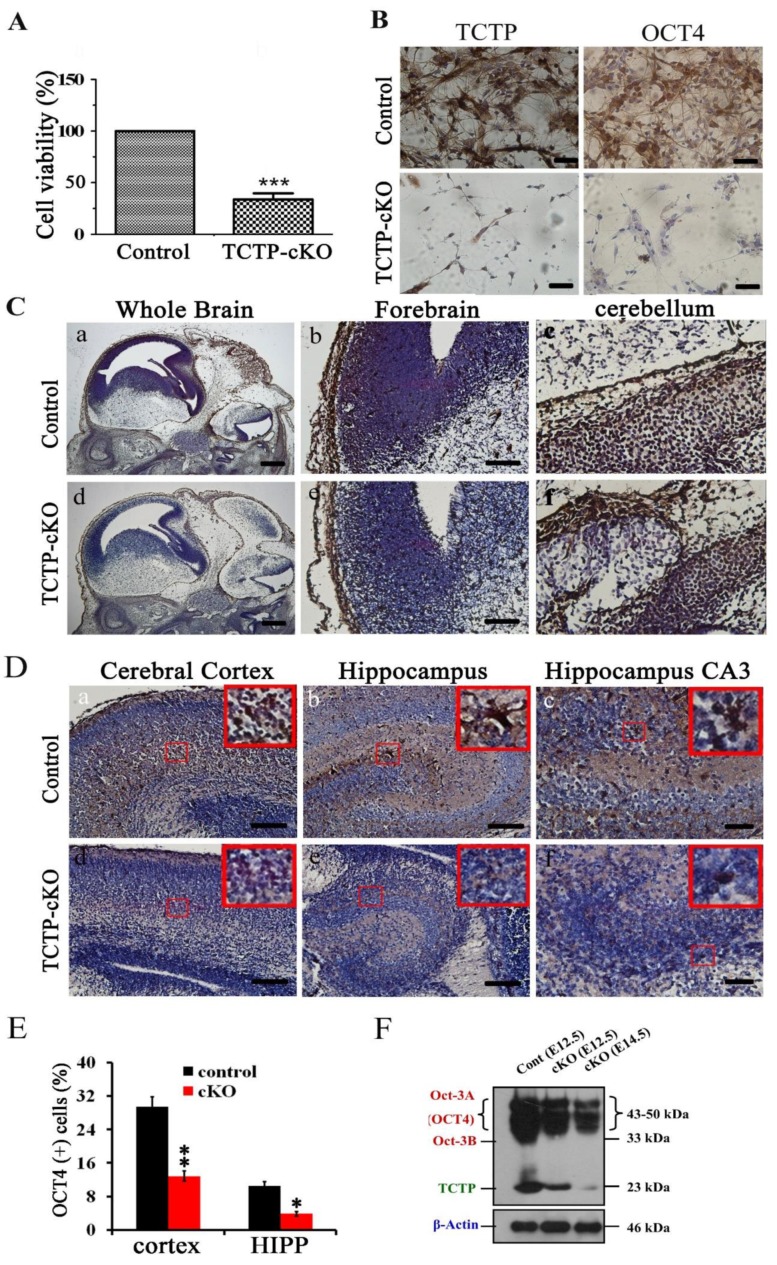
Cell viability and octamer-binding transcription factor 4 (Oct4) protein expression were detected in primary cortical progenitor cultures and brain sections. (**A**) Cell viability and cell number of neuron progenitor cell cultures from TCTP-deficient mice and littermate controls at E15.5 were detected by MTT assay. (*** *p <* 0.001, Student’s *t* test, *n* = 3 per group). (**B**) TCTP and Oct4 expression was detected by immunohistochemistry with antibody followed by DAB staining and then counterstaining with hematoxylin. (**C**,**D**) Oct4 expression of control and TCTP-cKO mice at E13.5 and P0.5 was detected by immunohistochemistry. A representative picture is shown for two independent experiments. (**E**) Bar graph summarizing quantification of Oct4-positive (+) cells in (**D**), (Data presented as mean ± SEM, * *p <* 0.05, ** *p <* 0.01, Student’s *t* test, *n* = 3 per group). (**F**) Protein level of Oct4 in control and TCTP-cKO mice was measured by Western blotting. Scale bar: A, 40 μm; C: a, d, 400 μm, b, c, e, f, 100 μm; D: a, b, d, e, 100 μm, c, f, 40 μm.

To further confirm whether neural precursor cell death observed in vivo and in vitro is caused by a cell autonomous or nonautonomous mechanism, we cultured primary cortical progenitor cells and measured their survival in vitro as an indicator of cell survival using MTT assay. Cortical progenitor cell cultures from *Nestin-cre*-mediated TCTP mutant embryos at E15.5 did not exhibit significant differences in cell aggregates, dendrite outgrowth, and differentiation at 24 h compared with littermate controls in vitro. After 3 days of culture, decreased cell viability and cell numbers were observed in TCTP-deficient cells (Figure 9A). Quantification of total cells per aggregation revealed a significant four-fold reduction in TCTP-deficient cultures, indicating that the cell death from *Nestin-cre*-derived TCTP disruption is caused by a cell autonomous mechanism.

**Figure 10:**
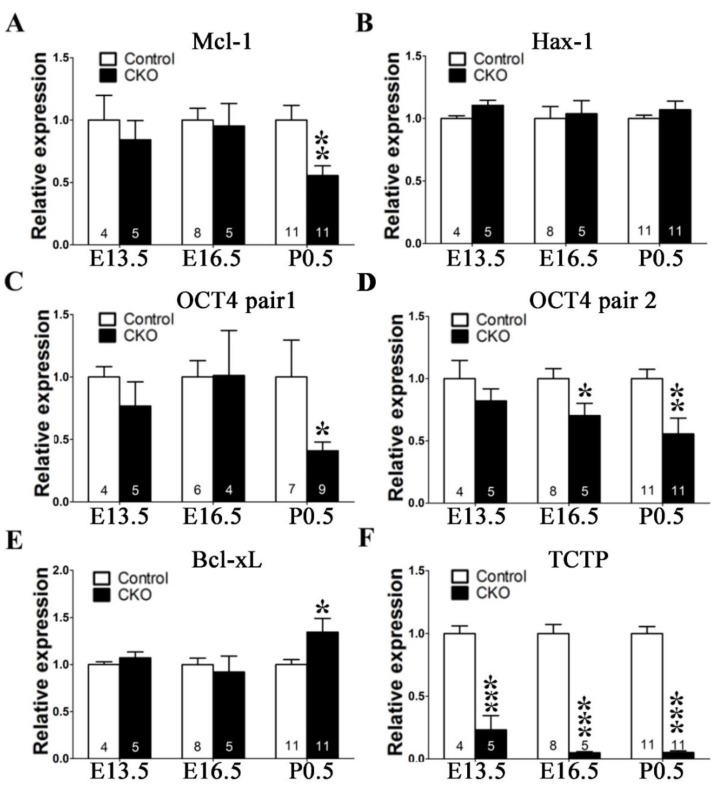
mRNA expression pattern of cell survival-related genes of TCTP-cKO mice compared with littermate controls. (**A**–**F**) Mcl-1, Hax-1, Oct-4 pair1, Oct-4 pair2, Bcl-xL, and TCTP mRNA expression from control and TCTP-cKO mice at E13.5, E16.5, and P0.5 were detected by qPCR. (Data presented as mean ± SEM, * *p* < 0.05, ** *p <* 0.01, *** *p <* 0.001, Student’s *t* test, *n* = 4–11 per group).

Niwa et al. showed that a critical amount of octamer-binding transcription factor 3/4 (Oct3/4) is required to sustain stem cell self-renewal, and up- or downregulation induced divergent developmental programs [34]. In addition, a previous study showed that Tpt1 activates transcription through binding to the regulatory region of the mouse *Oct4* gene in transplanted somatic nuclei in *Xenopus laevis* oocytes [35]. To examine whether the self-renewal of TCTP mutant mouse cortical progenitor cells may encounter a problem in undifferentiated embryonic stem cells, TCTP-modulated Oct4 expression was examined. Cortical progenitor cells were cultured from mouse brain at E15.5 and stained with antibody for Oct4 after 3 days of culture. Oct4-positive cells were detected in control groups, but a decrease was observed in the *Nestin-cre*-derived TCTP-deficient cell culture (Figure 9B). Moreover, we found significantly fewer cells and failure of differentiation of neuron dendrites in cortical progenitor cell culture. These results indicated that the loss of TCTP decreased cell numbers, suppressed transcription factor Oct4 expression, and disrupted cell differentiation in cortical progenitor cells cultured from *TCTP* mutant. The decreased Oct4 protein expression of TCTP-cKO mice was further confirmed in vivo by immunohistochemistry at E13.5 (Figure 9C) and P0.5 (Figure 9D,E) and Western blotting as early at E12.5 (Figure 9F) compared with littermate controls. Furthermore, we also studied the mRNA expression of Oct4 and cell survival-related genes with specific primers (Table 2). A significant decrease in Oct4 and Mcl-1 but not hax-1 and Bcl-xL mRNA in TCTP-cKO mice relative to control mice was observed (Figure 10). We expect that cells of cKO mice are needed for higher antiapoptotic activity, such as Bcl-xL and mcl-1, by the enhancement of mRNA expression levels. Bcl-xL protein was decreased, but Bcl-xL mRNA was increased in cKO mice at P0.5 (Figure 8C and Figure 10E). On the other hand, mcl-1 was decreased in both protein and mRNA levels at P0.5 (Figure 8C and Figure 10A).

Taken together, our results demonstrate that TCTP is essential for cell survival during early neuronal and glial differentiation during CNS development. Thus, enhanced neuronal and functional loss and decreased cell numbers of Tuj1 and doublecortin-positive neurons mediated through Mcl-1, Bcl-xL, Oct4, and cyclin D2 and E2 suppression and caspase-3 cleavage activation resulting in increased apoptosis and decreased proliferation may contribute to the perinatal death of *TCTP* mutant mice.

## 4. Discussion

In this study, we generated and characterized mutant mouse strains with selective deletion of TCTP in neural precursor cells mediated by *Nestin*-driven Cre expression. We found (1) a dramatic phenotype of growth retardation, (2) early postnatal death, (3) enhanced neuronal loss and functional defect on Tuj1 and doublecortin-positive neurons, (4) increased apoptosis and decreased proliferation, and (5) decreased expression of mMcl-1, Bcl-xL, hax-1, and Oct4 in *Nestin-cre*-driven TCTP conditional knockout mice. Furthermore, we also demonstrated an obligatory role of TCTP in the maturation process of neuronal progenitor cell development.

In prenatal development, neurogenesis is responsible for populating the growing brain. New neurons are continually made throughout adulthood in predominantly two regions of the brain, the subventricular zone (SVZ) lining the lateral ventricles, where new cells migrate to the olfactory bulb via the rostral migratory stream, and the subgranular zone (SGZ), part of the dentate gyrus of the hippocampus. We observed high levels of TCTP protein expression in those regions in wild-type mice at P0.5. Moreover, loss of TCTP protein in the brain caused decreased BrdU-positive cells in neurogenetic regions, and the size of the lateral ventricle of the mutant telencephalon was significantly larger relative to littermate controls at P1. These results suggest that TCTP regulates prenatal brain development and is involved in new neuron proliferation. Importantly, TCTP has been shown to bind to Mcl-1 and Bcl-xL, antiapoptotic members of the Bcl-2 family [9,36,37]. TCTP is therefore closely associated with apoptotic processes. The TUNEL-positive apoptotic cell-prone nature of the TCTP-deficient brain was first noted at E13.5 and significantly increased at E16.5, with maximal apoptotic cells found at P0.5. We showed that loss of TCTP expression dramatically decreased Mcl-1 and Bcl-xL protein expression then increased cleaved caspase-3 protein levels in apoptotic cells and during neurogenesis on postnatal day 0.5. This phenotype, together with the proliferation defect observed in TCTP mutants, is sufficient to explain why the TCTP-null mice died early during neurogenesis. These results are consistent with the finding from the study of *Xenopus laevis* [21], which demonstrated that TCTP regulates retinal axon growth through the interaction with Mcl-1 and Bcl-xL protein to inhibit caspase-3 activation. Our study demonstrated in a mammalian model system that TCTP is also important for whole brain development and that this function is essential for the perinatal survival of mice. We also found that loss of TCTP in the brain caused a significant decrease in Hax-1 protein expression. Previous studies indicated that Hax-1 was involved in the regulation of apoptosis or programmed cell death [38]. Hax-1 and TCTP have been reported to regulate calcium homeostasis. Homozygous deletion of Hax-1 in mice results in excessive apoptosis of neurons and postnatal death caused by a loss of motor coordination and function, leading to a failure to eat or drink [38]. These phenotypes mimic TCTP mutant mice, suggesting that TCTP might interact with Hax-1 directly or indirectly.

On the other hand, Susini et al. proposed that TCTP could anchor the mitochondria and bind to MCL-1 and Bcl-xL to inhibit the dimerization of Bax and protect from apoptotic cell death by antagonizing the Bax function [33]. However, the antiapoptotic function of TCTP occurs in the mitochondria, where it inhibits Bax-induced damage. Our data did not show a significant difference in the Bax protein level with whole-cell lysates when TCTP was deleted. An analysis with a purified fraction of mitochondria in the brain tissue is needed to substantiate our results and clarify this issue. There is Mcl-1 pro-survival large isoform (Mcl-1L) and pro-apoptotic short isoform (Mcl-1S) in cells. We detected Mcl-1L but not Mcl-1S. Therefore, a more suitable antibody and well-prepared cell lysates are needed to clarify the changes of Mcl-1L and Mcl-1S in cKO mice. In addition, p53 may contribute to the cell death induced by *TCTP* deletion. Recent evidence has demonstrated that TCTP can facilitate Mdm2-mediated ubiquitination of P53 by competing with and preventing the binding of Numb, an inhibitor of Mdm2, to the Mdm2-P53 complex [39]. Further study is needed to clarify the role of P53 in the defect CNS development of *TCTP* mutant mice. Our study exploring the role of p53 in neurodevelopmental defects of *TCTP* mutant mice with double knockout mice (*Nestin^Cre^*^/+^; *TCTP^flox^*^/*flox*^; *p53^−^*^/*−*^) is in progress. Our unpublished data showed that *P53* deficiency caused a more severe phenotype of *GFAP^Cre^*^/+^; *TCTP^flox^*^/*flox*^ knockout mice with *GFAP^Cre^*^/+^; *TCTP^flox^*^/*flox*^; *p53^−^*^/*−*^ double knockout mice. In addition, Hsu et al. demonstrated that dTCTP functions upstream of mTOR-dS6K and regulates fly cell growth by positively regulating dRheb activity [40]. Our previous publication showed that the mTOR-dS6K pathway contributed to the regulation of TCTP in beta cells (17) but not fibroblasts (15) in a cell-type-specific manner.

In this study, we showed that TCTP is required for the proliferation, differentiation, and migration of neuronal progenitor cells. The *Nestin-cre*-derived TCTP disruption specific to neuronal progenitor cells caused the loss of Tuj1- or DCX-negative cells (Figure 5) and cell death in TCTP-negative cells (Figure 8) mediated through a cell autonomous mechanism. In contrast, it has been shown that TCTP could be secreted through the TSAP6 protein, which is also involved in exosome production [8]. Secreted TCTP also promotes liver regeneration and enhances colorectal cancer invasion, suggesting that TCTP protein functions as a cell nonautonomous regulator of cancer cell growth and proliferation [41,42]. Therefore, we could not exclude that the loss of TCTP in neuronal progenitor cells induces cell death through cell autonomous and nonautonomous mechanisms simultaneously.

A previous study showed that decreased levels of Oct-4 resulted in a failure to form the inner cell mass and cell number, lost pluripotency, and differentiated into troph-ectoderm [43,44]. Therefore, the level of Oct-4 expression in mice is vital for regulating pluripotency and early cell differentiation, including the brain, spinal cord, and nervous system, since one of its main functions is to keep embryonic stem cells from differentiating [44]. Oct-4 is also involved in the self-renewal of undifferentiated neuronal stem cells by forming a heterodimer with Sox2 followed by binding to DNA [45]. Low Oct-4 expression sustains self-renewal but is deficient in differentiation [46].

Our in vitro results showed that a deficiency of TCTP, specifically in neuronal progenitor cells, led to decreased survival of neuron progenitor cells and reduced Oct-4 protein expression in vivo, suggesting that disruption of TCTP reduces the capability of Oct-4 to regulate the self-renewal of neuron progenitor cells. Our results suggest that TCTP deficiency in neuronal progenitor cells leads to defects in transcription factor signaling, particularly impaired Oct-4 activation. This result is consistent with the finding that TCTP activates transcription of Oct-4 in *Xenopus laevis* oocytes [35] and kidney-derived stem cells [47] but not in mouse embryonic carcinoma P19 cells and J1 embryonic stem cells in vitro [48]. These results imply that Oct-4 may co-interact with TCTP protein and facilitate neuron cell differentiation. Furthermore, TCTP may mediate Oct-4-stimulated dependent or independent signals in neural stem cells (NSCs). The deficiency of TCTP causes the inhibition of NSC self-renewal with unidentified signaling pathways.

On the other hand, our previous study indicated that homozygous deletion of TCTP reduced cyclins D2 and E2 in the cell cycle G1 and S phases in developing embryos [15]. This result is consistent with selective deletion of TCTP in the brain, suggesting that TCTP plays a role in embryonic and brain cell proliferation via a mechanism that is linked to the regulation of the cell cycle machinery. More experiments will be required to address the molecular mechanisms involved in this interesting finding.

There was little or no milk in the stomachs of TCTP-deficient mice a few hours after birth. Previous studies indicated that the inability to feed resulted in neonatal death caused by the absence of nourishment but also because the liquid derived from milk is essential for homeostatic processes in newborns [49]. Our data show that TCTP deficiency triggering neonatal death was not rescued by oral administration of saline containing 15% glucose (Supplementary Table S1). Our data show that there was no difference in the cardiac function detected by an echocardiogram with a VisualSonics Vevo 660TM high-resolution imaging system between TCTP-deficient mice (*Nestin-cre*; *TCTP^f^*^/^*^f^*) and littermate controls at E16.5 (unpublished results). Thus, the phenotype of early perinatal lethality of TCTP mutant mice was not caused by the heart problem. We also could not exclude the possibility that respiration failure may exist from the TCTP deficiency-induced defect in the respiration center in the pons and medulla. Several reports indicated that analysis of several neonatal lethal mutant mice revealed that nonfeeding or normal fasting newborns died 12 to 24 h after birth [49,50,51]. The TCTP mutant mice died between 24 and 36 h, suggesting that the inability to suckle milk was one possible cause of neonatal death. Feeding problems may be due to rejection by the mother. The major cause of the inability to feed in TCTP mutant mice might be associated with neuromuscular dysfunction.

Loss of TCTP in the developing brain might contribute to interference with normal physiological functions, such as the ability to suckle milk, olfactory sensitivity, movement coordination, and muscular function. Suckling is a complex process that consists of many structures of the brain and nerves, as well as the muscles required to extract milk [52]. According to our observation, TCTP-deficient mice can find and attach to a female mouse nipple, but we are not convinced whether these mice have a suckling response. The suckling response includes nipple attachment, suckling with rhythmic movement of the jaw and tongue, and the stretch response [53]. The interactions between motor and sensory neuronal pathways are linked to the central nervous system through the brain stem trigeminal complex. The data from Supplementary Figure S3 do not show a significant difference in the brain stem trigeminal ganglion between control and cKO mice detected by hematoxylin and eosin staining. To further confirm that the neurodevelopmental defects and phenotype of TCTP-cKO mice were due to the TCTP deficiency, *Nestin^Cre^*^/+^; *TCTP^f^*^/+^ mice will be crossed with hTCTP transgenic mice to produce double transgenic mice (*Nestin^Cre^*^/+^; *TCTP^f^*^/+^; *R26R-hTCTP*), which do not express the mouse TCTP protein but overexpress human TCTP, specifically in neuron progenitor cells. The phenotypes of the double transgenic mice will be compared with TCTP-cKO mice and *R26R-hTCTP* transgenic mice to check the rescue efficiency of hTCTP overexpression. Cortical neurons [25] and cortical progenitor cells [26] will be cultured for further study to explore whether disruption of TCTP sensitizes the neurons to apoptosis induced by DNA damage. On the other hand, Mcl-1 is a key regulator of apoptosis during CNS development [18]. *Nestin-cre*-derived knockout of TCTP decreased the expression of mMcl-1 (Figure 8C). Human Mcl-1 overexpression might rescue the early neonatal death of TCTP-cKO mutant mice.

Our results show new findings on the requirement for TCTP in the development and maintenance of neurons within the CNS. In vivo and in vitro results demonstrated that TCTP is required for neurogenesis and survival of the neuron maturation process. Additionally, TCTP regulates apoptotic cell death. Most importantly, our results implicate TCTP as a key regulatory molecule, involved in expanding the neural precursor pool and maintaining neuronal survival in the newborn mouse brain.

## 5. Conclusions

In our mouse model, TCTP deficiency in neuronal progenitor cells resulted in impaired neuronal and glial cell survival and differentiation through increased apoptosis and decreased proliferation, which led to perinatal death. We propose that TCTP is essential for cell proliferation, survival, and differentiation during CNS development. TCTP may be a target for the research of whole brain development and perinatal survival through the protection of neural precursor cells and neurons from apoptosis.

## Figures and Tables

**Table 1:** Genotype analysis of offspring from *Nestin^C^^re^*^/+^; *TCTP^f^*^/+^ mice crossed with *TCTP^f^*^/*f*^ mice.

Stage	Embryonic Time Point							
	E9.5~E10.5	E12.5	E14.5	E16.5	P0.5	P1.5	P3.5	P10.5
Litter	6	4	5	4	11	5	8	11
Total number of Offspring	37	22	31	33	73	38	48	71
Nestin^Cre/+^; TCTP^f/f^	10 (27%)	5 (23%)	8 (27%)	7 (21%)	25 (34%)	7 (18%)	0 (0%)	0 (0%)
Nestin^Cre/+^; TCTP^f/+^	12 (32%)	5 (23%)	10 (32%)	9 (27%)	25 (34%)	16 (42%)	16 (33%)	28 (39%)
Nestin^+/+^; TCTP^f/f^	5 (14%)	8 (36%)	6 (19%)	9 (27%)	14 (19%)	6 (16%)	19 (40%)	22 (31%)
Nestin^+/+^; TCTP^f/w^	10 (27%)	4 (18%)	6 (19%)	8 (24%)	9 (12%)	9 (24%)	13 (27%)	21 (30%)

**Table 2:** Primer sequences for qPCR gene expression analysis.

Sequence		
Gene	5′-Sense-3′	5′-Anti-Sense-3′
*Gapdh*	CGACTTCAACAGCAACTCCCACTCTTCC	TGGGTGGTCCAGGGTTTCTTACTCCTT
*Mcl-1*	GGAAGTCCTCGCCTGCGTCA	AAACATGGTCGGACGCCGCA
*Bcl-xL*	AGGCAGGCGATGAGTTTGAA	CGGCTCTCGGCTGCTGCATT
*Hax-1*	GACCTTGCCTTCCCACTCTCCTGA	GTCCCTGCGACCCCCAATCTG
*OCT4 pair1* *	GTGAGCCGTCTTTCCACCAGG	GGGTGAGAAGGCGAAGTCTG
*OCT4 pair2* ^#^	CCCTCCCTGGGGATGCTGTGAG	GAGTGACAGACAGGCCAGGCTCC
*Nanog*	TGCGGCTCACTTCCTTCTGACTTC	GGCCCTTGTCAGCCTCAGGAC
*TCTP*	TATATGAGGTTGGGGAGCGCCCG	CCTCCGGACCTTCAGCGGAA

* The pair of primers are located on exon 1 of OCT4 gene. ^#^ The pair of primers are located on exon 4 of OCT4 gene.

## Supplementary Materials

The following are available online at https://www.mdpi.com/2073-4409/9/1/133/s1.
