# Supplementary material for: TCTP is Essential for Cell Proliferation and Survival during CNS Development
Source: Cells. 2020 Jan 6;9(1):133. doi: 10.3390/cells9010133 (PMC7017002; doi:10.3390/cells9010133)
Supplement: Supplementary file 1 [file cells-09-00133-s001.pdf]

Supplementary Methodology

Whole Mount In Situ Hybridization

Whole mount in situ hybridization of mouse embryos was carried out essentially as described by Shen et al. (Supplementary Reference 1). The riboprobes for TCTP corresponding to the entire open reading frame of the cDNA were synthesized with a DIG RNA labeling kit (Roche, Indianapolis, IN, USA).

Supplementary Figure S1

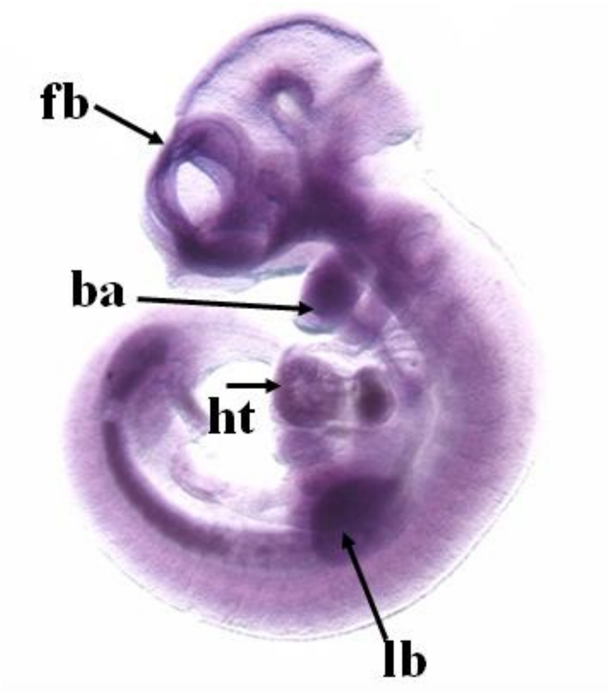

Supplementary Figure S1. TCTP RNA expression pattern at mouse embryonic stage 10 (E10.5). Whole-mount in situ hybridization from wild-type mice at E10.5 was carried out using riboprobes specific to *TCTP*. ba, branchial arch; fb, forebrain; ht, heart; lb, limb.

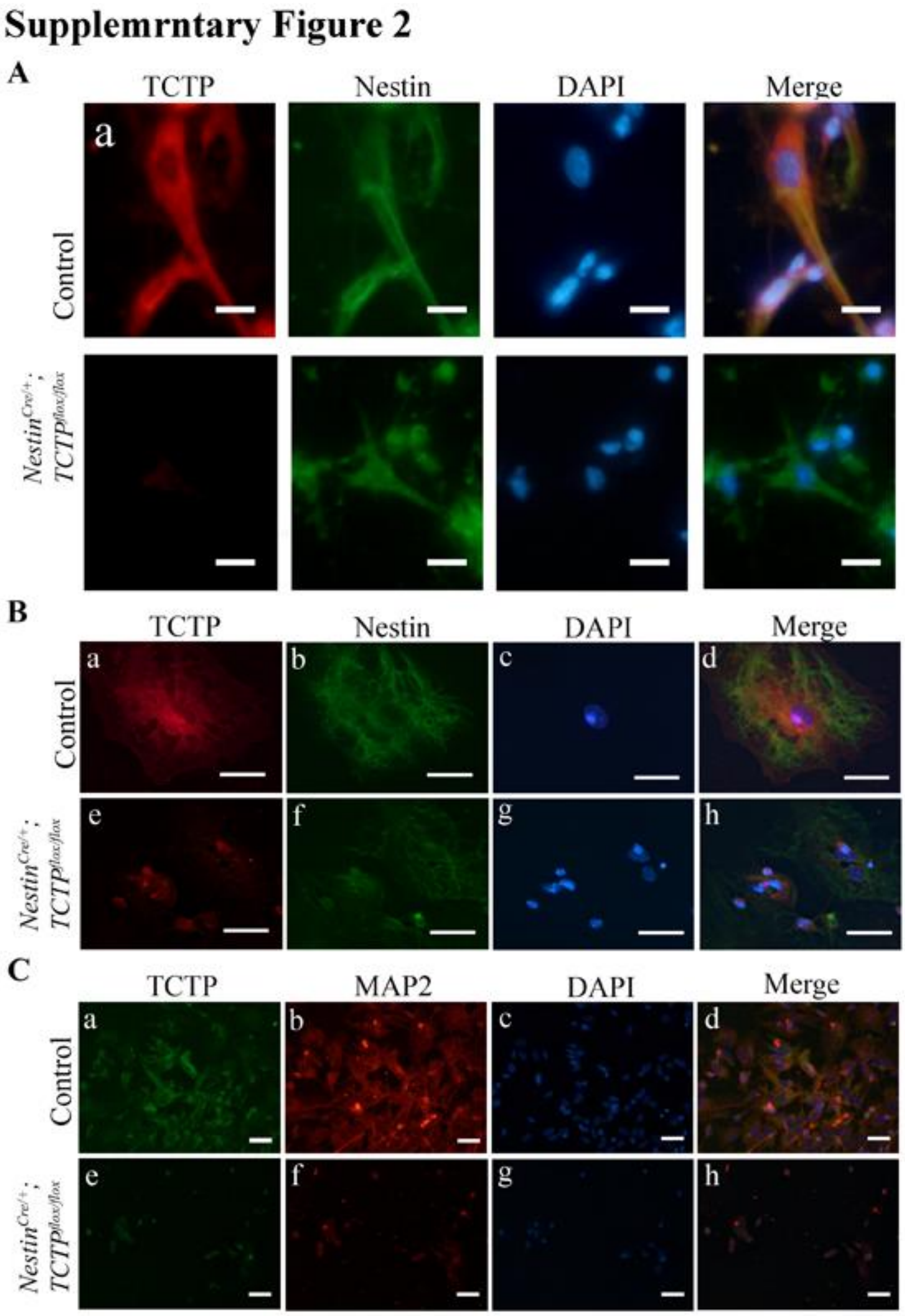

Supplementary Figure S2. Expression of proliferating neural precursor (Nestin^{+}) and postmitotic neuron (MAP2^{+}) markers was detected during critical period of brain development. Double immunofluorescence staining for TCTP (red) and Nestin (green) in primary culture from control and TCTP-cKO mice at (**A**) E16.5 and (**B**) E14.5. (**C**) Double immunofluorescence staining of MAP2 (red) and TCTP (green) in primary culture from control and *Nestin*$^{Cre/+}$; *TCTP*$^{flox/flox}$ mice at E16.5. Scale bars: A, 10 µm; B, C, 20 µm.

Supplementary Figure 3

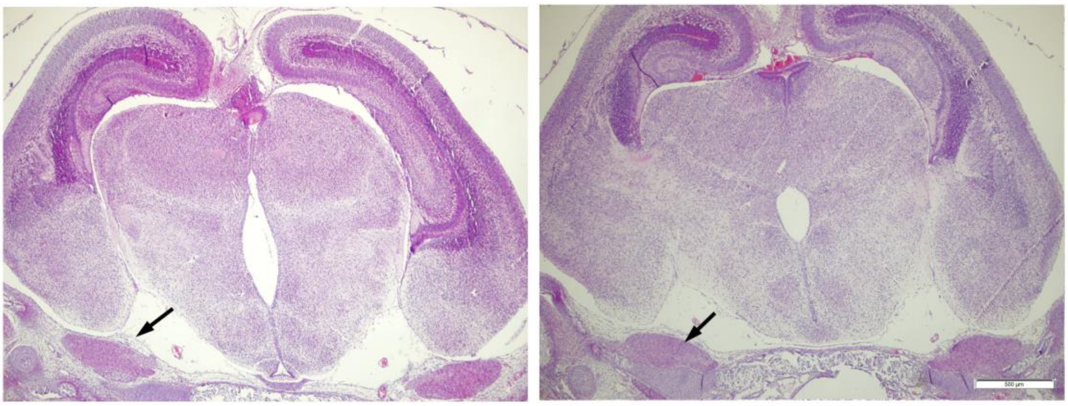

Control cKO

Supplementary Figure S3. Morphology of brain stem trigeminal ganglion was detected by hematoxylin and eosin stain. Coronal brain sections from control and TCTP cKO pups at E16.5 were stained with hematoxylin and eosin. Scale bars: 500 µm. Arrow indicates trigeminal ganglion.

Supplementary Table S1

TCTP-deficient mice were not rescued by oral administration of 0.2 mL saline containing 15% glucose 2 times per day.

Supplementary Table 1

Genotype	Time (h) Post administration									
	3h	6h	9h	18h	21h	24h	36h	39h	43h	52h
$Nestin^{cre/+}$; $TCTP^{f/f}$	9	9	8	7	4	4	3	3	3	0
$Nestin^{cre/+}$; $TCTP^{f/+}$	13	13	13	12	12	12	12	11	11	11
$Nestin^{+/+}$; $TCTP^{f/f}$	7	7	7	7	7	7	7	7	7	7
$Nestin^{+/+}$; $TCTP^{f/+}$	8	8	8	8	8	8	8	6	6	6

Supplementary references

1. Shen, M.M.; Wang, H.; Leder, P. A differential display strategy identifies Cryptic, a novel EGF-related gene expressed in the axial and lateral mesoderm during mouse gastrulation. *Development (Cambridge, England)* **1997**, *124*, 429-442.
